# A Nutritional Conditional Lethal Mutant Due to Pyridoxine 5′-Phosphate Oxidase Deficiency in *Drosophila melanogaster*

**DOI:** 10.1534/g3.114.011130

**Published:** 2014-04-15

**Authors:** Wanhao Chi, Li Zhang, Wei Du, Xiaoxi Zhuang

**Affiliations:** * Department of Neurobiology, University of Chicago, Chicago, Illinois 60637; † Department of Ecology and Evolution, University of Chicago, Chicago, Illinois 60637; ‡ Ben May Department for Cancer Research, University of Chicago, Chicago, Illinois 60637

**Keywords:** nutritional conditional lethal, auxotrophs, pyridoxine 5′-phosphate oxidase, vitamin B6, congenital metabolic diseases

## Abstract

The concept of auxotrophic complementation has been proposed as an approach to identify genes in essential metabolic pathways in *Drosophila melanogaster*. However, it has achieved limited success to date, possibly due to the low probability of finding mutations fit with the chemically defined profile. Instead of using the chemically defined culture media lacking specific nutrients, we used bare minimum culture medium, *i.e.*, 4% sucrose, for adult *Drosophila*. We identified a nutritional conditional lethal mutant and localized a c.95C > A mutation in the *Drosophila pyridoxine 5′-phosphate oxidase* gene [*dPNPO* or *sugarlethal* (*sgll*)] using meiotic recombination mapping, deficiency mapping, and whole genome sequencing. PNPO converts dietary vitamin B6 such as pyridoxine to its active form pyridoxal 5′-phosphate (PLP). The missense mutation (*sgll*^95^) results in the substitution of alanine to aspartate (p.Ala32Asp). The *sgll*^95^ flies survive well on complete medium but all die within 6 d on 4% sucrose only diet, which can be rescued by pyridoxine or PLP supplement, suggesting that the mutation does not cause the complete loss of PNPO activity. The *sgll* knockdown further confirms its function as the *Drosophila* PNPO. Because better tools for positional cloning and cheaper whole genome sequencing have made the identification of point mutations much easier than before, alleviating the necessity to pinpoint specific metabolic pathways before gene identification, we propose that nutritional conditional screens based on bare minimum growth media like ours represent promising approaches for discovering important genes and mutations in metabolic pathways, thereby accelerating the establishment of *in vivo* models that recapitulate human metabolic diseases.

*Drosophila melanogaster* has been proven to be a valid model system for studying human genetic diseases, such as developmental disorders, cancer, and neurological disorders (Bier 2005). Recently, an increasing number of metabolic studies have been performed for *D. melanogaster* (Birse *et al.* 2010; Celotto *et al.* 2006; Esslinger *et al.* 2013; Liao *et al.* 2010; Liu *et al.* 2012; Matzkin *et al.* 2011; Morris *et al.* 2012; Na *et al.* 2013; Reed *et al.* 2010; Subramanian *et al.* 2013; Ugrankar *et al.* 2011; Wightman *et al.* 2012; Zhang *et al.* 2009). Many *Drosophila* metabolic mutants that model aspects of human diabetes, obesity, or other metabolic diseases have been established (Baker and Thummel 2007; Liu and Huang 2013). Nevertheless, compared to the wide array of human congenital metabolic diseases, which have been increasingly discovered in recent years (Boycott *et al.* 2013), valid *in vivo* models are still largely lacking.

One particular type of metabolic mutant is termed auxotrophic mutants or auxotrophs (Davis and Mingioli 1950). Due to the loss of capacity to synthesize critical metabolic end products, auxotrophs will not proliferate or survive in the defined diet or the culture media lacking specific nutrients unless they are supplemented with the missing nutrients or related substances. Genetic screens based on such a concept have been successfully performed for bacteria (Frank 1963; Frisch *et al.* 1991; Leon-Del-Rio *et al.* 1995; Payne *et al.* 1977) and yeast (Ma *et al.* 2007; Pronk 2002; Stolz *et al.* 1999) and have helped to identify key enzymes in specific metabolic pathways.

A similar concept was proposed for fly genetics in 1969 by Vyse and Nash (1969). They exposed Oregon-R wild-type flies to mutagen ethyl methane-sulphonate and selected mutants with yeast-sucrose medium lacking RNA. Although wild-type *Drosophila* could survive in such defined minimum culture media, one mutant line was identified because it required purine and pyrimidine supplements (Vyse and Sang 1971). However, the combination of the power of fly genetics and such a concept has had very limited success to date (Adachi-Yamada *et al.* 1999; Falk and Nash 1974; Kunte *et al.* 2006; O’Donnell *et al.* 2000). The advantage of such a design is that the chemically specific supplement helps to define the impaired pathway in the mutant. However, the chances of identifying mutations that fit with the chemically defined profile are small.

Here we describe the identification of a pyridoxine 5′-phosphate oxidase (PNPO) hypomorphic *D. melanogaster* mutant line (*sgll^95^*) based on the nutritional conditional lethal phenotype. PNPO deficiency in humans has very severe consequences, including neonatal epileptic encephalopathy (NEE) (Bagci *et al.* 2008; Hoffmann *et al.* 2007; Khayat *et al.* 2008; Mills *et al.* 2005; Ruiz *et al.* 2008). Different from the earlier chemically specific designs, we used 4% sucrose as our minimum growth medium (wild-type flies can survive for at least 6 d under such a condition), therefore casting a wide net for potential nutritional conditional mutants. Because better tools for positional cloning and cheaper whole genome sequencing have made the identification of point mutations much easier than before, alleviating the necessity to pinpoint specific metabolic pathways before gene identification, we reason that the nutritional conditional screens based on bare minimum growth medium like ours represent promising approaches for discovering important genes and mutations in metabolic pathways, thereby accelerating the establishment of *in vivo* models that recapitulate human metabolic diseases.

## Materials and Methods

### *Drosophila melanogaster* strains

The *w^1118^* were used as wild-type controls. *Ddc-GAL4* flies (genotype: *w^1118^*; *P{w[+mC]=Ddc-GAL4.L}4.36*; Bloomington *Drosophila* Stock #7009) were backcrossed onto *w^1118^* background for five generations. Unless otherwise stated, all *Drosophila* were reared on standard corn meal medium made at University of Chicago. *CG31472* UAS-RNAi transgenic line, as well as its control, was obtained from the Vienna Drosophila RNAi Center (VDRC) (Dietzl *et al.* 2007). The GAL4 driver (genotype: y[1] w[*]; *P{w[+mC]=Act5C-Gal4}25FO1/Cyo*, *y[+]*) was obtained from Bloomington stock center (Stock #4414).

### Survival study under various conditions

A group of 20 male flies, 2 to 4 d old, were anesthetized briefly and transferred into a culture vial filled with 4% sucrose in 1% agar (sugar-only diet), 5% yeast in 1% agar, or 5% yeast plus 4% sucrose in 1% agar. Survival was checked daily. The survival rate was calculated from four vials per condition.

### Measuring consumption with a spectrophotometry method

To quantify the amount of food intake, a group of 20 male flies were starved overnight in a culture vial supplied with water only. Flies were then allowed to feed on a 3% agar plate containing 4% sucrose and 1% FD&C blue No. 1 (Cat #FD110; Spectrum) for 30 min at room temperature in the dark. After feeding, the animals were homogenized in 500 μl PBS buffer centrifuged at 13,000 rpm for 25 min. The supernatant was centrifuged again before being transferred to cuvettes. Absorbance was measured at 630 nm. A separate group fed on dye-free plates was used so that the absorbance measured from the dye-free group was subtracted from the absorbance measured from the blue dye group. The net absorbance reflected the amount of food ingested. Results from six vials per condition were averaged.

### Measuring consumption with the capillary feeder assay

The assay setup was modified from Ja *et al.* (2007). Flies were individually housed during the capillary feeder (CAFE) assay with 5% yeast in 1% agar provided on the bottom of the assay compartment and 4% sucrose provided in the capillary. The addition of the 5% yeast in this particular assay was to ensure that the mutant flies would survive well. The consumption of each fly was recorded daily after flies were acclimated with the environment. Forty-eight flies were measured for each genotype. The data presented in this article were the averages from 3 d.

### Inverse PCR to recover the insertion points of *P*-element

Inverse PCR was performed by following the GDP_iPCR Protocol from the Gene Disruption Project (Bellen *et al.* 2004). Briefly, genomic DNA was extracted from the whole body (DNA extraction kit; Cat #D6015; Zymo Research) and cut with *Hin*p I. DNA fragments were ligated as PCR templates. A pair of primers EY.3.F and EY.3.R was used (http://flypush.imgen.bcm.tmc.edu/pscreen/files/GDP_iPCRProtocol_051611.pdf). The sequence of PCR product was blasted online against the *Drosophila melanogaster* genome sequence.

### Precise excision of the *P{w[+mC]=Ddc-GAL4.L}* insertion

The *P{w[+mC]=Ddc-GAL4.L}* insertion in the limpet gene was mobilized in flies heterozygous for the insertion and for a Kip-marked third chromosome carrying the Δ2-3 transposase. Males were selected and mated with virgin female TM3/TM6 flies to generate balanced excision lines. Individual white-eyed flies lacking Kip were selected and mated with the insertion line to generate heterozygous flies. Flies from each line were divided into two groups: one group was tested on 4% sucrose for phenotype and one group was used for preparing genomic DNA for PCR to validate the precise excision events. PCR primers were: 5′- GCA ACG CAA TGC ATT GCT AAT AA-3′ and 5′- ACC GTT CGG CAG CCG AAG TAG C-3′. The PCR product of the wild-type band is 240 bp.

### Whole genome sequencing, data analysis, and SNV detection

Genomic DNA was extracted (DNA extraction kit; Cat #D6015; Zymo Research). Whole genome sequencing was performed by the Genomics Core Facility at the University of Chicago. Raw reads were aligned to the genome (UCSC dm3 version) using bowtie2 (parameter:–very-sensitive-local) (Langmead and Salzberg 2012; Meyer *et al.* 2013). The bam files for target region 84D14 to 84E3 were extracted by samtools (Li *et al.* 2009). GATK (McKenna *et al.* 2010) was then used to identify variations in the target region. The resulting variation sites were manually checked in IGV (Robinson *et al.* 2011).

### Pyridoxine and PLP treatment

To get a stock solution (5 mg/ml), 100 mg of pyridoxine (Item #148277; Finest Nutrition) was dissolved into 20 ml H_2_O. Pyridoxal 5′-phosphate (PLP; Cat #82870; Sigma) was dissolved into 1 M HCl to get stock solution (50 mg/ml). The stock solution was then diluted with 4% sucrose in 1% agar to get different concentrations of vitamin B6–supplemented sugar diet; 4% sucrose without vitamin B6 was used as control. A group of 20 male flies, age 2 to 4 d, was anesthetized briefly and transferred into the vials. Survival was checked daily. The survival rate was calculated from three vials per condition.

## Results

### A mutant line exhibited a lethal phenotype on 4% sucrose

**Figure 1:**
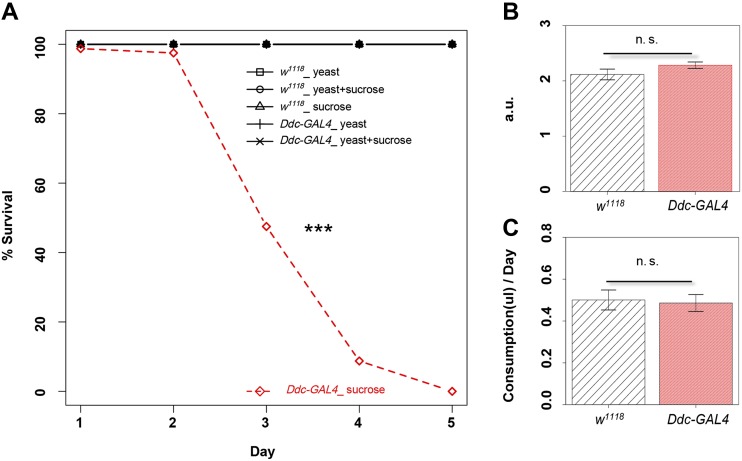
Lethal phenotype of *Ddc-GAL4* flies on sugar-only diet. (A) Survival of wild-type (*w^1118^)* and *Ddc-GAL4* flies on different diets. ***P < 0.001, log-rank test, comparing *Ddc-GAL4* flies on sugar-only diet to each of other groups. (B) The *w^1118^* and *Ddc-GAL4* flies consumed similar amounts of food by a colorimetric method. (C) The *w^1118^* and *Ddc-GAL4* flies consumed similar amounts of food by CAFE assay. a.u., arbitrary unit; n.s., not significant; unpaired *t* test. Error bars indicate ± SEM.

During a study of the role of dopamine in *Drosophila* feeding behavior, we observed that many homozygous, but not heterozygous, *Ddc-GAL4* flies could not survive on 4% sucrose-only diet for more than 3 d, whereas most wild-type flies could survive for more than 10 d. Sugar lethal phenotype was observed in both female and male *Ddc-GAL4* flies. To rule out the possibility that the genetic background contributed to the phenotype, we crossed this line with *w^1118^*, the control strain used in this study, for five generations by following the mini-white marker. Backcrossed homozygous *Ddc-GAL4* flies still exhibited the lethal phenotype on 4% sucrose but they survived well on 5% yeast or 5% yeast plus 4% sucrose (Figure 1A). We quantified the survival rate of adult male flies on 4% sucrose-only diet. Although all homozygous *Ddc-GAL4* flies died within 6 d, no *w^1118^* control flies died within 6 d (Figure 1A). However, *Ddc-GAL4* and control flies had similar consumption, measured with both a colorimetric method (Figure 1B) and a dynamic measurement of food intake, the CAFE assay (Ja *et al.* 2007) (Figure 1C). Taken together, we conclude that the diet-dependent lethal phenotype of *Ddc-GAL4* flies is not due to the impaired feeding or due to the sucrose toxicity; it is most likely caused either by their deficient sugar catabolism or by their oversensitivity to the lack of specific nutrients in 4% sucrose-only diet.

### The lethal phenotype is not caused by the *P*-element insertion

To determine if the conditional lethal phenotype is due to the gene disruption caused by *P*-element insertion in *Ddc-GAL4* flies, we first performed inverse PCR. Sequencing of the inverse PCR products revealed that the *P*-element was inserted into intron 4 of the *limpet* gene on the third chromosome. We then performed precise excision by crossing the *Ddc-GAL4* line with the Δ2-3 transposase line (see *Materials and Methods* for details). Precise excision was confirmed by sequencing the PCR products amplified by a pair of primers flanking the insertion site. The flies with precise excision were then subjected to sugar-only diet. We found that their lethal phenotype on sugar-only diet was not rescued by precise excision, demonstrating that another gene was responsible for the phenotype. We therefore named this gene *sugarlethal* (*sgll*).

### Meiotic recombination mapping localized the mutation to a small region on chromosome 3

Recombination mapping was performed based on the fact that flies homozygous for the mutant allele are 100% lethal, whereas those heterozygous for the mutant allele are 0% lethal within 6 d on 4% sucrose-only diet (Supporting Information, Figure S1). We first crossed the sgll* line with a meiotic mapping line that carries the following recessive markers: *ru*, *h*, *th*, *st*, *cu*, *sr*, *e*, and *ca* (genotype: *ru[1] h[1] th[1] st[1] cu[1] sr[1] e[s] ca[1]*; Bloomington *Drosophila* Stock #576). Female F1 flies were then crossed with a second meiotic mapping line (genotype: *ru[1] h[1] th[1] st[1] cu[1] sr[1] e[s] Pr[1] ca[1]/TM6B,Bri[1],Tb[1]*; Bloomington *Drosophila* Stock #1711) that carries the same recessive markers as well as a dominant marker *Pr* (Prickly). The resulting *Pr*^+^ male flies with various combinations of recessive markers were individually crossed with the sgll* line. *Pr*^−^ flies from that cross were subjected to the 4% sucrose assay. Among the 80 lines we screened, flies from 36 lines survived completely well on 4% sucrose and flies from 44 lines did not. We compared the survival phenotype and the presence or the absence of each genetic marker in the corresponding male breeder. The results are summarized in Table 1. We calculated recombination events that were indicated either by “death” phenotype and positive for the respective marker or by “survival” phenotype and negative for the respective marker. The recombination fraction between the mutation and each of the four genetic markers (*h*, *th*, *cu*, and *e*) is (8+13)/80 (26.25%), (0+2)/80 (2.5%), (1+2)/80 (3.75%), and (2+10)/80 (15%), respectively. Therefore, the mutation most likely locates between *th* (thread arista; cytogenetic locus 72C1-72D1) and *cu* (curled wings; 86D7-86D7).

**Table 1:** Number of flies in each category in the recombination mapping study

	Death		Survival	
Markers	Negative	Positive	Negative	Positive
*h*	36	8	13	23
*th*	44	0	2	34
*cu*	43	1	2	34
*e*	42	2	10	26

### Deficiency mapping localized the mutation to a 100 kbp region

**Figure 2:**
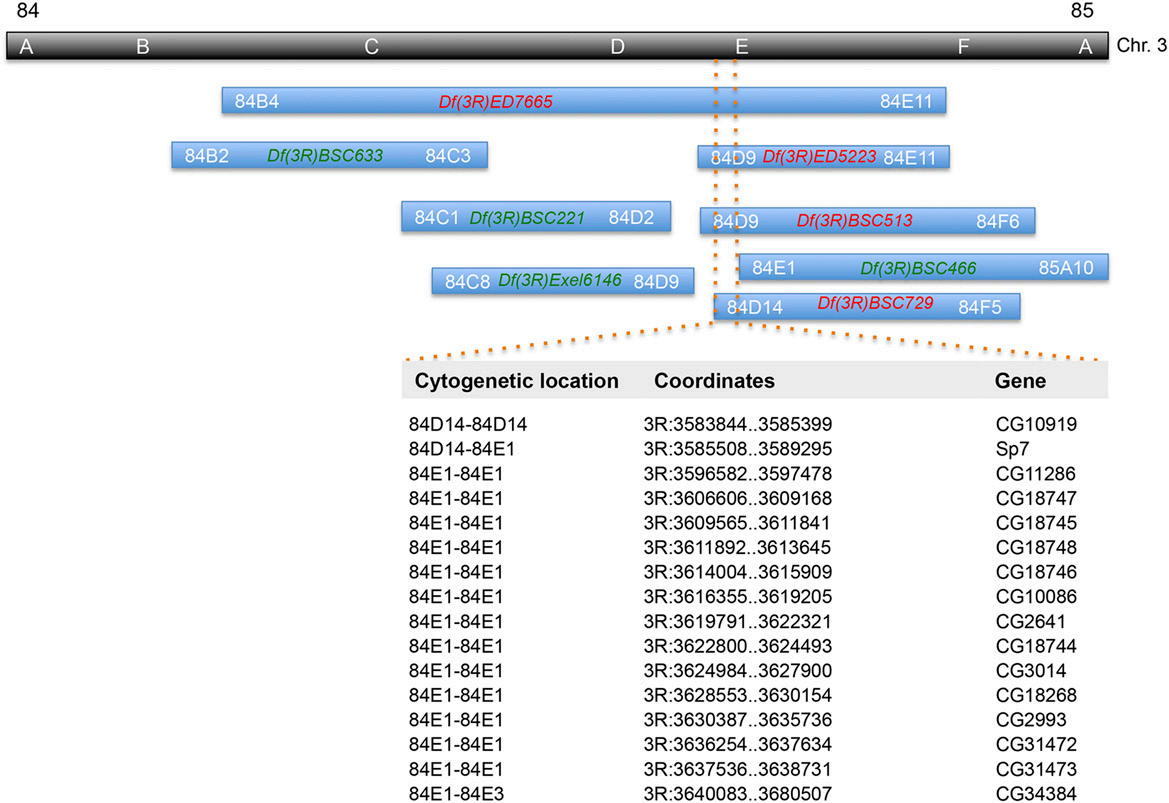
Deletion alignments of deficient lines. The blue bar indicates the chromosome segment deletion covered in deficient lines. Each bar is labeled by its line name. A red name indicates the heterozygous flies derived from this line die on 4% sucrose-only diet. A green name indicates survival. After comparing sequence coordinates of these deficient lines, we narrowed the causative gene to ∼100-kbp region in the genome that contains 16 genes in Flybase.

We next performed the deficiency mapping by crossing the sgll* line with deficient lines with various deletions of DNA segments within the region of interest (Figure S2). Deficiency (Df) /sgll* heterozygous flies were subjected to the 4% sucrose assay. Our reasoning was that if flies could not survive on 4% sucrose-only diet, then the mutation of interest is located within the region deleted in the deficient line. A total of 29 deficient lines covering a region from 71D3 to 86C7 were tested (Table S1). All flies except the ones derived from *Df(3R)ED7665* survived on 4% sucrose. In addition, we noticed that the number of Df/sgll* flies derived from *Df(3R)ED7665* were much fewer than expected [7 Df/sgll* flies out of the total of 124 (7+117)] (Table 2). We suspected that these Df/sgll* flies could not survive well even on the standard fly food. To test that, newly emerged Df/sgll* flies were transferred to a new vial with the standard fly food. All died after approximately 1 d. The difference in severity of phenotype between Df/sgll* flies and the sgll* homozygotes suggest that the mutation in the sgll* line might cause a partial loss of function. In *Df(3R)ED7665*, the region from 84B4 to 84E11 is deleted. To further narrow the region of interest, seven more deficient lines that cover 84B4 to 84E1 were tested (Table S1). Among them, three showed similar results as *Df(3R)ED7665* did (Table 2). The rates of Df/sgll* are 1/85, 9/88, and 10/91 for *Df(3R)ED5223*, *Df(3R)BSC513*, and *Df(3R)BSC729*, respectively. By comparing the sequence coordinates, we finally localized the causative gene to 84D14–84E1 on the cytogenetic map. This region spans ∼100 kilobase pairs (kbp) in the genome and contains 16 genes in Flybase (Marygold *et al.* 2013) (Figure 2).

**Table 2:** Number of adult flies in each genotype generated from deficiency lines crossed with the sgll* line

Deficiency Line	Df/sgll*	Balancer/sgll*
*Df(3R)ED7665*	7	117
*Df(3R)ED5223*	1	84
*Df(3R)BSC513*	9	79
*Df(3R)BSC729*	10	81

Df, deficiency.

### Identification of a c.95C > A mutation in *CG31472* in the sgll^*^ line by whole genome sequencing

**Figure 3:**
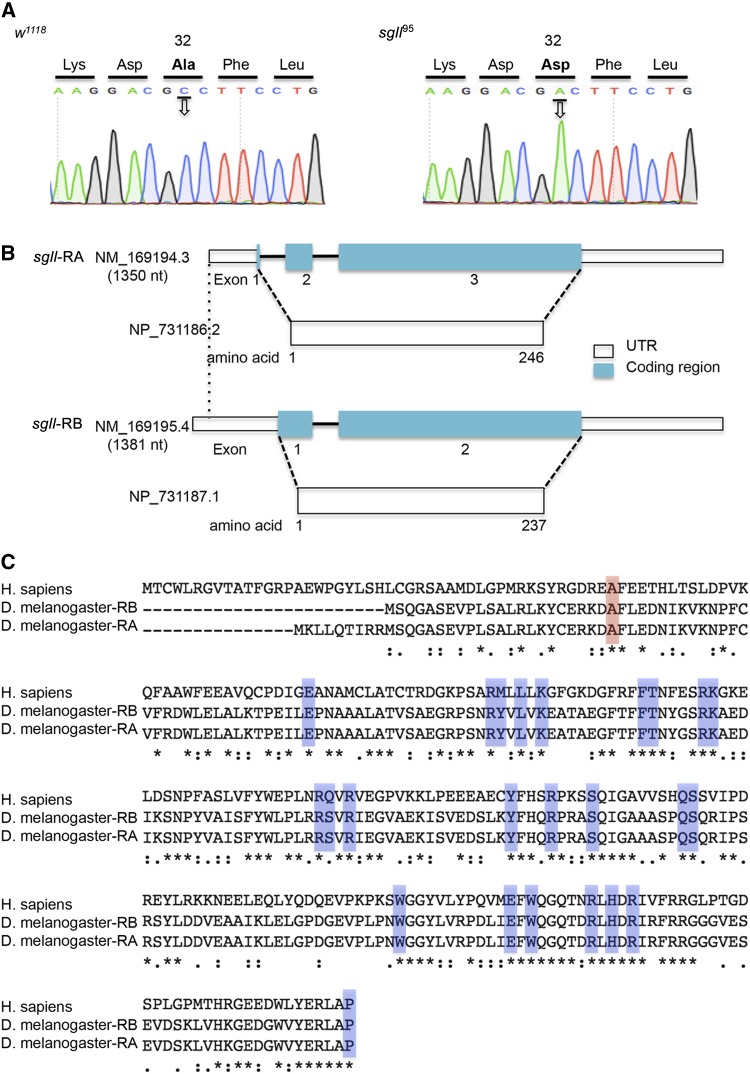
Mutation, gene structure, and homology analysis. (A) A homozygous missense mutation in *sgll* was validated by targeted Sanger sequencing. (B) Gene structure of *sgll*. (C) Amino acid sequence alignment analysis. Known essential residues for enzyme activity are shaded in blue. The alanine at +32 is highlighted with red. Human PNPO amino acid sequences were obtained from NCBI (RefSeq:NP_060599.1). Semi-conserved (.), conserved (:); and absolutely conserved (*) are shown.

To identify the causative gene and the mutation, whole genome sequencing was performed. Genomic DNA from the backcrossed sgll* line and *w^1118^* were extracted from the whole body. Each genomic DNA sample was then sequenced on a sequencer as paired-end 100 bp reads, with 400× coverage (Illumina HI-SEQ2000). Filtered reads were aligned to the *Drosophila* genome reference sequence (Dm3) with bowtie2, allowing detection of single-nucleotide variants (SNVs). Sequencing data revealed three SNVs in these 16 genes between *w^1118^* and the sgll* line. Among them, two SNVs are located in the intron region of *CG34384*, which less likely disrupts the gene function. However, the third SNV is located in the exon region of *CG31472*, which was further validated by target Sanger sequencing of PCR products using DNA templates isolated from *w^1118^* and the sgll* line (Figure 3A). The homozygous c.95C > A substitution in the exon 2 of *CG31472*, designated *sgll*^95^, results in the substitution of a neutral alanine to an acidic aspartate (p.Ala32Asp) in the protein sequence (Figure 3A).

### Gene structure and homology analysis of *sgll*

The *sgll* spans a region of ∼1.4 kbp in the genome (RefSeq: NM_169194.3 and NM_169195.4). It encodes two putative PNPO proteins due to alternative transcriptional starts. These two proteins have the same C-terminal sequence with one variant containing nine extra amino acid residues at the N-terminus (Figure 3, B and C).

Amino acid sequence alignment analysis exhibited more than 75% similarity (more than 40% identity) between SGLL and human PNPO (Figure 3C). The alanine at +32 is also conserved between humans and flies. Crystal structure studies of human PNPO showed that some amino acid residues were critical to the enzyme activity (Musayev *et al.* 2003). In the alignment analysis, we found that almost all these critical residues are conserved (Figure 3C). Moreover, the C-terminus that is essential for the enzyme activity is also highly conserved between humans and flies (Kang *et al.* 2004) (Figure 3C).

### Lethal phenotype of mutant flies in 4% sucrose was rescued by both pyridoxine and pyridoxal 5′-phosphate

**Figure 4:**
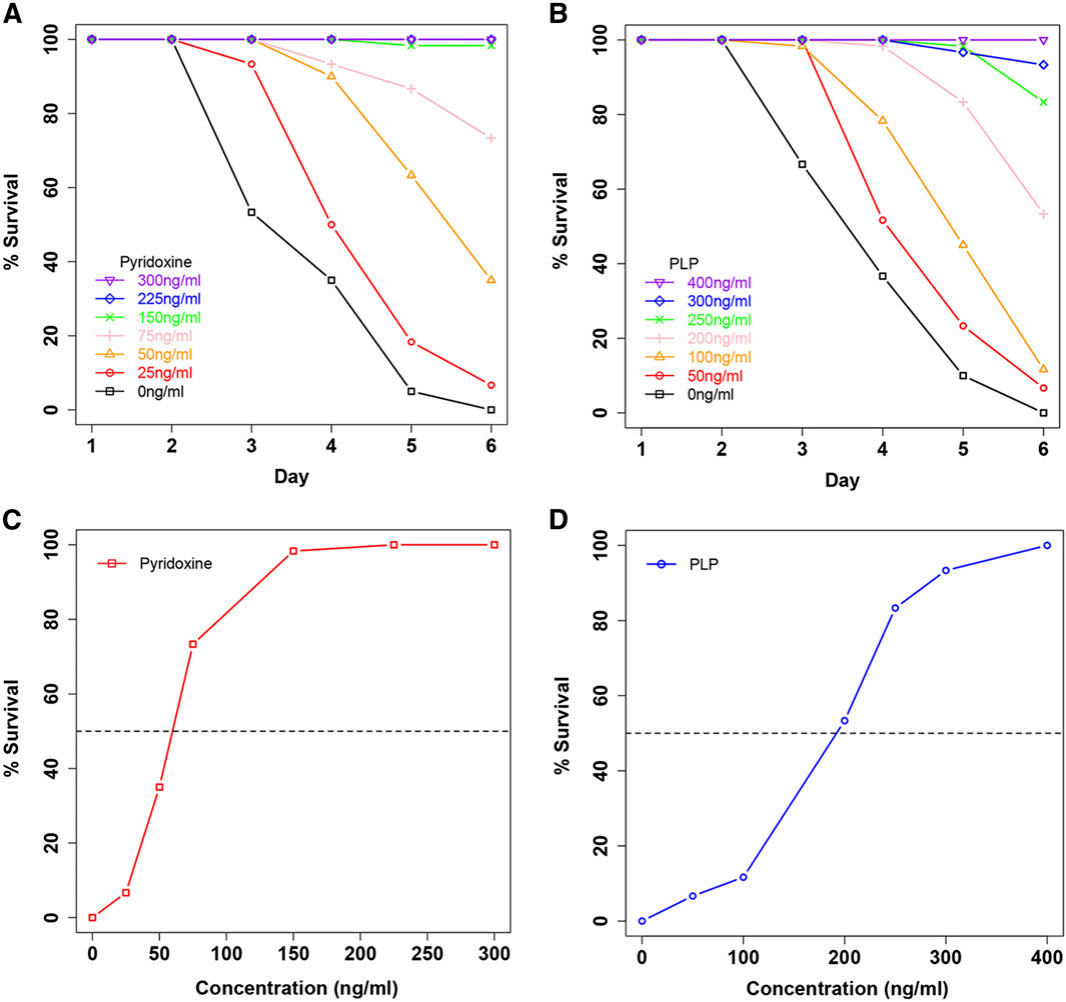
Pyridoxine and PLP supplement of sugar-only diet rescued the lethal phenotype of *sgll*^95^ flies. Pyridoxine and PLP were added to 4% sucrose and survival was checked daily for pyridoxine groups, PLP groups, and the 4% sucrose-only group. Compared with 100% death in 4% sucrose-only group by day 6, flies with pyridoxine supplement (A) and PLP supplement (B) survived better. Dose-response curves were plotted based on the survival data on day 6 (C, D).

PNPO is a rate-limiting enzyme in converting inactive vitamin B6 in the diet to PLP, the only active form of vitamin B6. Complete PNPO deficiency is lethal in mice (Skarnes *et al.* 2011). The fact that the *sgll*^95^ homozygotes survive well on the standard fly food suggests that the p.Ala32Asp mutation in SGLL only leads to a partial loss of its function. We therefore reasoned that both the inactive and the active forms of vitamin B6 supplement should reduce the lethal phenotype of the mutant on sugar-only diet. To test this hypothesis, homozygous *sgll*^95^ flies were picked into vials with 4% sucrose and six different concentrations of pyridoxine or PLP. Compared with 100% death in 4% sucrose-only group by day 6, both pyridoxine and PLP supplement increased the survival of the flies in a dose-dependent manner (Figure 4, A and B). Therefore, we conclude the following: SGLL is the *Drosophila* homolog of PNPO; the conditional lethal phenotype of *sgll*^95^ flies is caused by the oversensitivity to the lack of vitamin B6 in the diet; and c.95C > A mutation in SGLL only partially decreases PNPO enzyme activity.

### Sugar lethal phenotype was also observed in *sgll* knockdown flies, which was rescued by PLP, but not pyridoxine

To further confirm the role of *sgll* deficiency in causing lethality of *sgll*^95^ flies on 4% sucrose-only diet, we used RNAi to knockdown the expression of *sgll*. Quantitative PCR results revealed that *sgll* was knocked down at least 90% by Act5C-driven expression of the RNAi construct in both females and males (data not shown). To test if knockdown flies were responsive to pyridoxine and PLP treatment, 1-d-old flies were picked into vials with one of three conditions: 4% sucrose; 4% sucrose plus 75 ng/ml pyridoxine; and 4% sucrose plus 200 ng/ml PLP. To compare pyridoxine and PLP, the concentrations of pyridoxine and PLP were chosen based on their EC50 in their respective dose-response curves in rescuing *sgll*^95^ flies (Figure 4, C and D). At such relatively low concentrations, all knockdown flies died within 3 d. However, the survival of knockdown flies within 24 hr was significantly rescued by PLP but not pyridoxine (Table 3), suggesting that PNPO activity in knockdown flies is too low to convert pyridoxine to PLP.

**Table 3:** Survival rates of *sgll* knockdown flies after being fed under different conditions for 24 hr

Treatment	*Act5C-GAL4*/RNAi	*Act5C-GAL4*/*wt*	RNAi/*wt*
Control	0.58	1	1
Pyridoxine	0.53	1	1
PLP	0.84*	1	1

N = 34–40 per group.

* *P* < 0.05, chi-square test of homogeneity.

## Discussion

We report the identification of a c.95C > A mutation in *CG31472* in *Drosophila melanogaster* that leads to the identification of the *Drosophila* homolog of *PNPO*. The missense mutation results in the substitution of a neutral alanine to an acidic aspartate (p.Ala32Asp) in amino acid sequence. Such a mutation leads to an accelerated lethal phenotype in diet devoid of vitamin B6, which can be rescued by dietary supplement of either pyridoxine or PLP.

PNPO is a rate-limiting enzyme in converting inactive vitamin B6 in the diet to PLP, the only active form of vitamin B6. Pyridoxine and pyridoxamine are inactive forms of dietary vitamin B6 (Garrido-Franco 2003). They are first phosphorylated by kinase and then oxidized by PNPO to PLP. Unlike bacteria and plants, insects and mammals cannot synthesize vitamin B6 in a *de novo* manner; therefore, dietary vitamin B6 is essential for survival. The fact that pyridoxine can rescue the lethal phenotype of *sgll*^95^ homozygotes on sugar-only diet suggests that the p.Ala32Asp mutation only leads to a partial loss of PNPO enzyme function such that it can convert enough pyridoxine to PLP. This is also supported by the survival of the mutants on standard fly food because all dietary vitamin B6 forms are inactive before being converted to PLP.

PNPO is highly conserved through species (Musayev *et al.* 2003). The protein product of *sgll* shares more than 75% similarity with human PNPO. In humans, PNPO deficiency can cause NEE. In NEE patients, severe seizures usually appear within hours of birth and are unresponsive to anticonvulsant drugs. NEE caused by severe *PNPO* deficiency does not respond to pyridoxine. However, in cases of partial *PNPO* deficiency, pyridoxine can ameliorate the seizures (Pearl *et al.* 2013). Other than seizures, patients with *PNPO* deficiency also exhibit metabolic disorders, such as hypoglycemia, acidosis, and anemia (Clayton *et al.* 2003; Mills *et al.* 2005) because PLP is a co-factor required for more than 100 enzymes that function in amino acid metabolism, gluconeogenesis, immune system, and neurotransmitter synthesis (Canzanello *et al.* 1995; Eliot and Kirsch 2004). Until now, we know little about exactly how *PNPO* deficiency causes NEE.

In humans, congenital metabolic diseases, also referred to as inborn errors of metabolism, are mostly rare genetic diseases due to mutations in single genes. Although rare, collectively they represent a large class of genetic diseases that have been increasingly discovered in recent years (Boycott *et al.* 2013). Congenital metabolic diseases usually lead to devastating consequences. Early diagnosis, such as prenatal genetic testing, is the key to early interventions, including dietary changes or nutritional supplements that could be life-saving. However, the diversity of such diseases and the small sample size of any particular disease have largely hampered our understanding of the underlying genetic defects and the affected systems. Animal models that recapitulate human conditions and have well-defined genetic basis and metabolic symptoms could offer the ideal tools needed in the field. In this work, we present an example of a potentially efficient approach using bare minimum growth media to identify nutritional conditional mutants in *Drosophila* and to discover genes and mutations that may cause congenital metabolic diseases.

## Supplementary Material

Supporting Information
