# Supplementary material for: A Nutritional Conditional Lethal Mutant Due to Pyridoxine 5′-Phosphate Oxidase Deficiency in Drosophila melanogaster
Source: G3 (Bethesda). 2014 Apr 15;4(6):1147–54. doi: 10.1534/g3.114.011130 (PMC4065258; doi:10.1534/g3.114.011130)
Supplement: Supporting Information [file supp_4_6_1147__index.html]

A Nutritional Conditional Lethal Mutant Due to Pyridoxine 5′-Phosphate Oxidase Deficiency in Drosophila melanogaster — Supporting Information 

# A Nutritional Conditional Lethal Mutant Due to Pyridoxine 5′-Phosphate Oxidase Deficiency in *Drosophila melanogaster*

## Supporting Information for Chi *et al.*, 2014

**Files in this Data Supplement:**

- Supporting Information - Figures S1-S2 and Table S1 (PDF, 581 KB)
- Figure S1 - Recombination mapping breeding and screening scheme. (PDF, 172 KB)
- Figure S2 - Deficiency mapping breeding and screening scheme. (PDF, 147 KB)
- Table S1 - Deficient lines used in this study. (PDF, 414 KB)
