# Supplementary material for: A Nutritional Conditional Lethal Mutant Due to Pyridoxine 5′-Phosphate Oxidase Deficiency in Drosophila melanogaster
Source: G3 (Bethesda). 2014 Apr 15;4(6):1147–54. doi: 10.1534/g3.114.011130 (PMC4065258; doi:10.1534/g3.114.011130)
Supplement: Supporting Information [file supp_g3.114.011130_011130SI.pdf]

G3: Genes | Genomes | Genetics

A nutritional conditional lethal mutant due to pyridoxine 5'-phosphate oxidase deficiency in *Drosophila melanogaster*

Wanhao Chi*, Li Zhang[§], Wei Du[†], Xiaoxi Zhuang*
*The Department of Neurobiology
[§]The Department of Ecology & Evolution
[†]The Ben May Department for Cancer Research
The University of Chicago, Chicago IL 60637

Corresponding author:
Xiaoxi Zhuang, Ph.D.
The University of Chicago
J.F Knapp Research Center, Room R214
924 E. 57th street, Chicago IL 60637
Tel: 773-8349063
Email: xzhuang@bsd.uchicago.edu

DOI: 10.1534/g3.114.011130

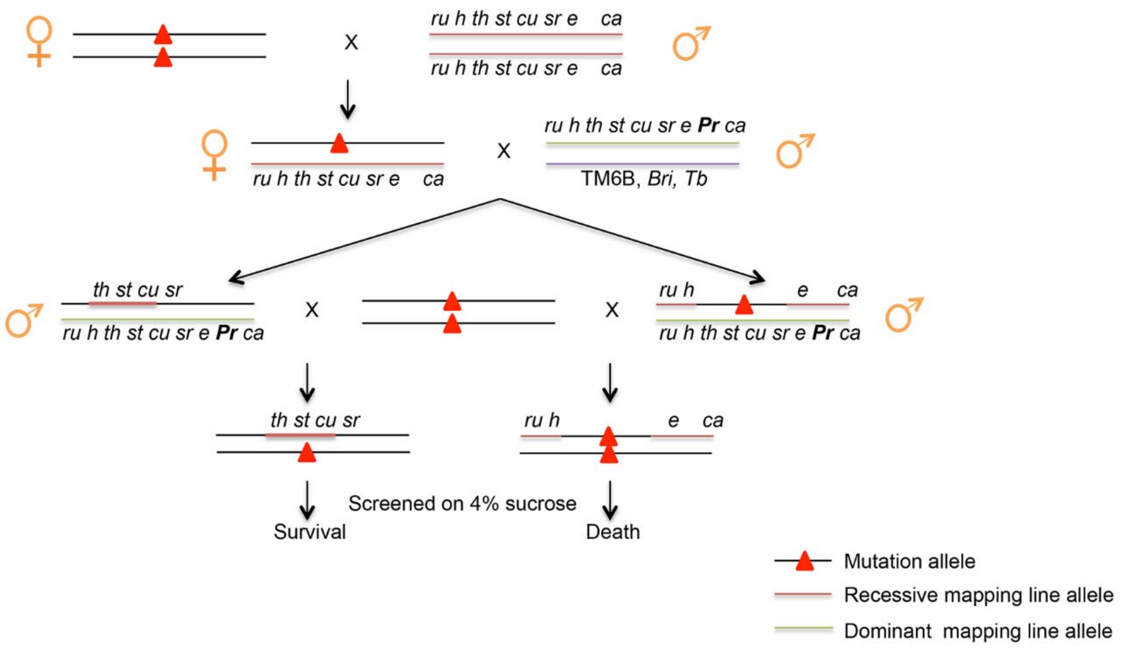

Figure S1 Recombination mapping breeding and screening scheme. Various recombinants of genetic markers were generated by breeding *sgll** flies with two mapping lines. Individual recombinant were subsequently bred with *sgll** flies to generate flies for phenotyping.

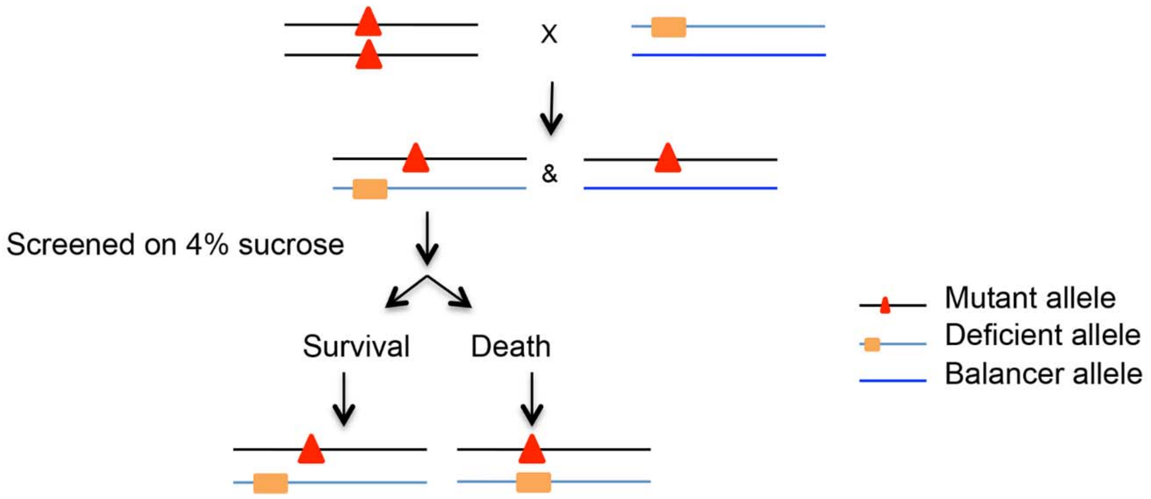

Figure S2 Deficiency mapping breeding and screening scheme. The *sgII** flies were bred with a deficient line. F1 flies without Balancer were subjected to 4% sucrose screening. Based on the phenotype, we could infer whether or not the causative gene was covered by the deficient line.

Table S1. Deficient lines used in this study. All lines were from Bloomington Drosophila Stock Center.

	Line #	Symbol	Genotype	Deleted Segment	Coordinates
The 1st screening	27888	Df(3L)BSC845	w[1118]; Df(3L)BSC845/TM6C, Sb[1] cu[1]	71D3;72A1	3L:15504128;15819023
	8078	Df(3L)ED4606	w[1118]; Df(3L)ED4606, P{w[+mW.Scer \FRT.hs3]=3'.RS5+3.3'}ED4606/TM6C, cu[1] Sb[1]	72D4;73C4	3L:16080584;16773223
	8098	Df(3L)ED4674	w[1118]; Df(3L)ED4674, P{w[+mW.Scer \FRT.hs3]=3'.RS5+3.3'}ED4674/TM6C, cu[1] Sb[1]	73B5;73E5	3L:16654384;17042518
	8099	Df(3L)ED4685	w[1118]; Df(3L)ED4685, P{w[+mW.Scer \FRT.hs3]=3'.RS5+3.3'}ED4685/TM6C, cu[1] Sb[1]	73D5;74E2	3L:16884176;17605270
	24948	Df(3L)BSC444	w1118; Df(3L)BSC444/TM6C, Sb1 cu1	74A5;75A7	3L:17346537;17871939
	27347	Df(3L)BSC775	w[1118]; Df(3L)BSC775/TM6C, Sb[1] cu[1]	75A2;75E4	3L:17788244;18891426
	9697	Df(3L)BSC220	w[1118]; Df(3L)BSC220/TM6C, Sb[1] cu[1]	75F1;76A1	3L: 18965662--18965925;19164368
	8087	Df(3L)ED229	w[1118]; Df(3L)ED229, P{w[+mW.Scer \FRT.hs3]=3'.RS5+3.3'}ED229/TM6C, cu[1] Sb[1]	76A1;76E1	3L:19163806;19995811
	6646	Df(3L)BSC20	Df(3L)BSC20, st[1] ca[1]/TM6B, Tb[1]	76A7--B1;76B4--5	
	3617	Df(3L)kto2	Df(3L)kto2/TM6B, Tb[+]	76B1--2;76D5	
	8088	Df(3L)ED4858	w[1118]; Df(3L)ED4858, P{w[+mW.Scer \FRT.hs3]=3'.RS5+3.3'}ED4858/TM2	76D3;77C1	3L:19888473;20394920
	27917	Df(3L)BSC839	w1118; Df(3L)BSC839/TM6C, Sb1 cu1	77B4;77C6	3L:20313247;20486308
	27369	Df(3L)BSC797	w[1118]; Df(3L)BSC797/TM6C, Sb[1] cu[1]	77C3;78A1	3L:20445923;20942833
	25116	Df(3L)BSC553	w[1118]; Df(3L)BSC553/TM6C, Sb[1]	78A2;78C2	3L: 20984731--20985064;21219092
	24923	Df(3L)BSC419	w[1118]; Df(3L)BSC419/TM6C, Sb[1] cu[1]	78C2;78D8	3L:21218032;21597878
	8101	Df(3L)ED4978	w[1118]; Df(3L)ED4978, P{w[+mW.Scer \FRT.hs3]=3'.RS5+3.3'}ED4978/TM6C, cu[1] Sb[1]	78D5;79A2	3L:21526907;21873785
	9700	Df(3L)BSC223	w[1118]; Df(3L)BSC223/TM6C, Sb[1] cu[1]	79A3;79B3	3L: 21909520--21909525;22078536
	24955	Df(3L)BSC451	w[1118]; Df(3L)BSC451/TM6C, Sb[1] cu[1]	79B2;79F5	3L: 22069195;22684788--22684831
	8089	Df(3L)ED230	w[1118]; Df(3L)ED230, P{w[+mW.Scer \FRT.hs3]=3'.RS5+3.3'}ED230/TM6C, cu[1] Sb[1]	79C2;80A4	3L:22127751;22827471
	9226	Df(3R)ED5100	w[1118]; Df(3R)ED5100, P{w[+mW.Scer \FRT.hs3]=3'.RS5+3.3'}ED5100/TM6C, cu[1] Sb[1]	81F6;82E7	3R:22995;912807
	8967	Df(3R)ED5147	w[1118]; Df(3R)ED5147, P{w[+mW.Scer \FRT.hs3]=3'.RS5+3.3'}ED5147/TM6C, cu[1] Sb[1]	82E7;83A1	3R:912842;1193526
	8965	Df(3R)ED5156	w[1118]; Df(3R)ED5156, P{w[+mW.Scer \FRT.hs3]=3'.RS5+3.3'}ED5156/TM6C, cu[1] Sb[1]	82F8;83A4	3R:1090655;1284574
	26533	Df(3R)BSC681	w1118; Df(3R)BSC681, P+PBac{XP3.RB5} BSC681/TM6C, Sb1 cu1	83E2;83E5	3R:2111067;2206257
	25077	Df(3R)BSC549	w[1118]; Df(3R)BSC549/TM6C, Sb[1]	83A6;83B6	3R:1328526;1442413
	7443	Df(3R)BSC47	Df(3R)BSC47, st[1] ca[1]/TM3, P{w[+m*]=Ubx-lacZ.w[+]}TM3, Sb[1]	83B7--C1;83C6--D1	
	1990	Df(3R)Tpl10	Df(3R)Tpl10, Dp(3;3)Dfd[rv1], kni[ri-1] Dfd[rv1] p[p] Doa[10]/TM3, Sb[1]	83C1--2;84B1--2	
	8685	Df(3R)ED7665	w[1118]; Df(3R)ED7665, P{w[+mW.Scer \FRT.hs3]=3'.RS5+3.3'}ED7665/TM6C, cu[1] Sb[1]	84B4;84E11	3R:2916249;3919805
	24970	Df(3R)BSC466	w1118; Df(3R)BSC466/TM6C, Sb1 cu1	84E1;85A10	3R:3657392;4573406
	9215	Df(3R)ED5495	w[1118]; Df(3R)ED5495, P{w[+mW.Scer \FRT.hs3]=3'.RS5+3.3'}ED5495/TM6C, cu[1] Sb[1]	85F16;86C7	3R:5996223;6712482
The 2nd screening	25724	Df(3R)BSC633	w1118; Df(3R)BSC633/TM6C, cu1 Sb1	84B2;84C3	3R:2906110;2949098
	7625	Df(3R)Exel6146	w[1118]; Df(3R)Exel6146, P{w[+mC]=XP-U} Exel6146/TM6B, Tb[1]	84C8;84D9	3R:2988409;3317319
	9698	Df(3R)BSC221	w[1118]; Df(3R)BSC221/TM6B, Tb[+]	84C1;84D2	3R:2933489;3037519
	24927	Df(3R)BSC423	w1118; Df(3R)BSC423/TM6C, Sb1 cu1	84D1;84D5	3R:3012954;3222044
	9076	Df(3R)ED5223	w[1118]; Df(3R)ED5223, P{w[+mW.Scer \FRT.hs3]=3'.RS5+3.3'}ED5223/TM6C, cu[1] Sb[1]	84D9;84E11	3R:3317426;3919805
	25017	Df(3R)BSC513	w1118; Df(3R)BSC513/TM6C, Sb1 cu1	84D9;84F6	3R:3356396;4076143
	26581	Df(3R)BSC729	w1118; Df(3R)BSC729, P+PBac{XP3.RB5} BSC729/TM6C, Sb1 cu1	84D14;84F5	3R:3575809;4069851
