# Supplementary material for: A Nutritional Conditional Lethal Mutant Due to Pyridoxine 5′-Phosphate Oxidase Deficiency in Drosophila melanogaster
Source: G3 (Bethesda). 2014 Apr 15;4(6):1147–54. doi: 10.1534/g3.114.011130 (PMC4065258; doi:10.1534/g3.114.011130)
Supplement: Supporting Information [file supp_g3.114.011130_FigureS1.pdf]

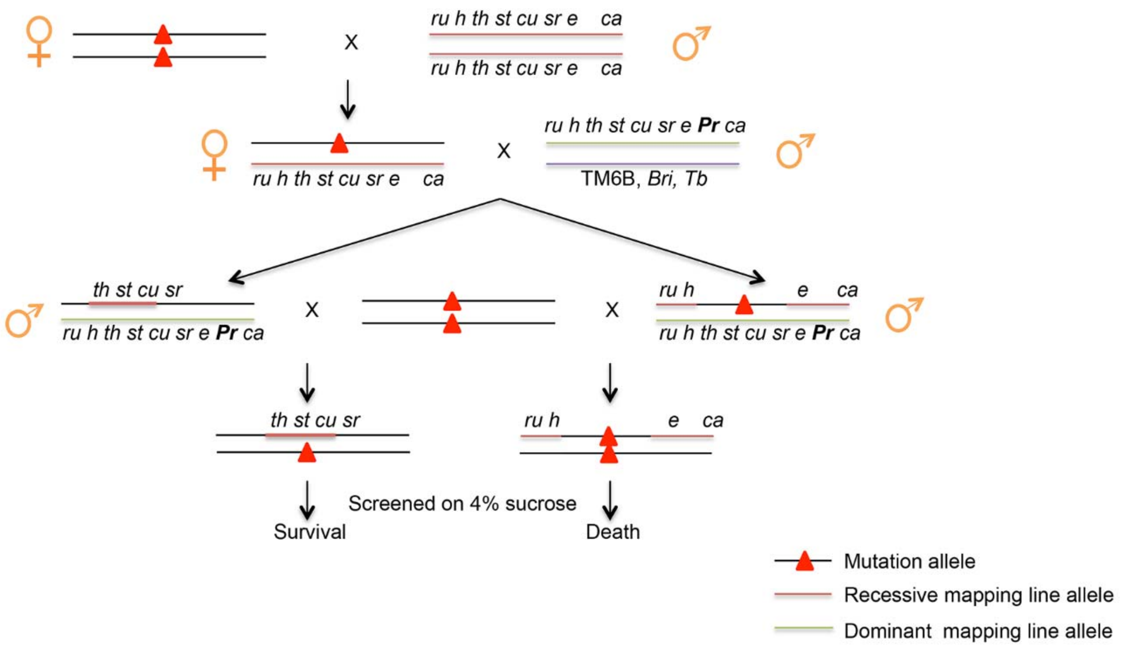

Figure S1 Recombination mapping breeding and screening scheme. Various recombinants of genetic markers were generated by breeding *sgll** flies with two mapping lines. Individual recombinant were subsequently bred with *sgll** flies to generate flies for phenotyping.
