# Supplementary material for: A Nutritional Conditional Lethal Mutant Due to Pyridoxine 5′-Phosphate Oxidase Deficiency in Drosophila melanogaster
Source: G3 (Bethesda). 2014 Apr 15;4(6):1147–54. doi: 10.1534/g3.114.011130 (PMC4065258; doi:10.1534/g3.114.011130)
Supplement: Supporting Information [file supp_g3.114.011130_FigureS2.pdf]

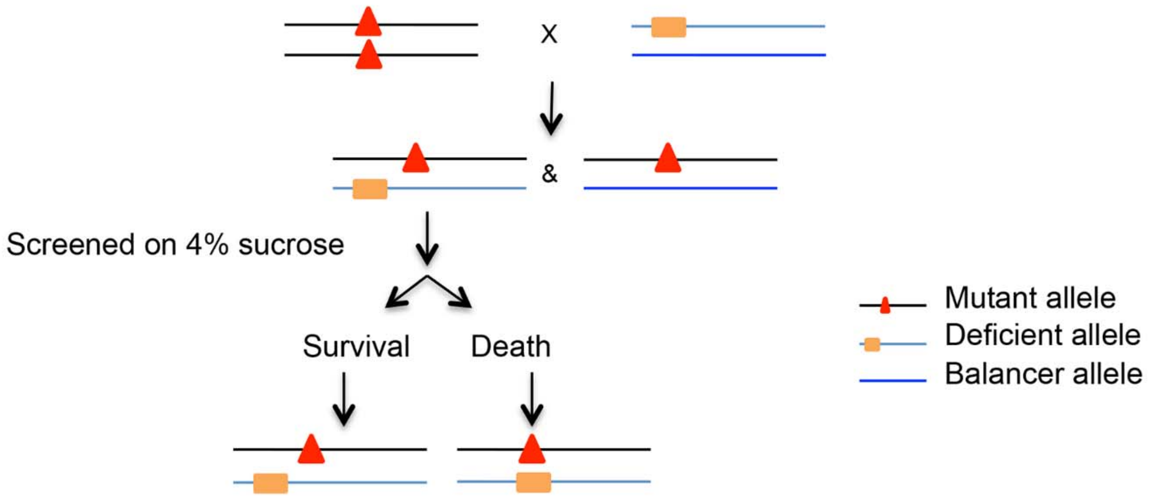

Figure S2 Deficiency mapping breeding and screening scheme. The *sgll** flies were bred with a deficient line. F1 flies without Balancer were subjected to 4% sucrose screening. Based on the phenotype, we could infer whether or not the causative gene was covered by the deficient line.
