# Supplementary material for: A Nutritional Conditional Lethal Mutant Due to Pyridoxine 5′-Phosphate Oxidase Deficiency in Drosophila melanogaster
Source: G3 (Bethesda). 2014 Apr 15;4(6):1147–54. doi: 10.1534/g3.114.011130 (PMC4065258; doi:10.1534/g3.114.011130)
Supplement: Supporting Information [file supp_g3.114.011130_TableS1.pdf]

Table S1. Deficient lines used in this study. All lines were from Bloomington Drosophila Stock Center.

| | Line # | Symbol | Genotype | Deleted Segment | Coordinates |
|---|---|---|---|---|---|
| The 1st screening | 27888 | Df(3L)BSC845 | w[1118]; Df(3L)BSC845/TM6C, Sb[1] cu[1] | 71D3;72A1 | 3L:15504128;15819023 |
| | 8078 | Df(3L)ED4606 | w[1118]; Df(3L)ED4606, P{w[+mW.Scer \FRT.hs3]=3'.RS5+3.3'}ED4606/TM6C, cu[1] Sb[1] | 72D4;73C4 | 3L:16080584;16773223 |
| | 8098 | Df(3L)ED4674 | w[1118]; Df(3L)ED4674, P{w[+mW.Scer \FRT.hs3]=3'.RS5+3.3'}ED4674/TM6C, cu[1] Sb[1] | 73B5;73E5 | 3L:16654384;17042518 |
| | 8099 | Df(3L)ED4685 | w[1118]; Df(3L)ED4685, P{w[+mW.Scer \FRT.hs3]=3'.RS5+3.3'}ED4685/TM6C, cu[1] Sb[1] | 73D5;74E2 | 3L:16884176;17605270 |
| | 24948 | Df(3L)BSC444 | w1118; Df(3L)BSC444/TM6C, Sb1 cu1 | 74A5;75A7 | 3L:17346537;17871939 |
| | 27347 | Df(3L)BSC775 | w[1118]; Df(3L)BSC775/TM6C, Sb[1] cu[1] | 75A2;75E4 | 3L:17788244;18891426 |
| | 9697 | Df(3L)BSC220 | w[1118]; Df(3L)BSC220/TM6C, Sb[1] cu[1] | 75F1;76A1 | 3L: 18965662--18965925;19164368 |
| | 8087 | Df(3L)ED229 | w[1118]; Df(3L)ED229, P{w[+mW.Scer \FRT.hs3]=3'.RS5+3.3'}ED229/TM6C, cu[1] Sb[1] | 76A1;76E1 | 3L:19163806;19995811 |
| | 6646 | Df(3L)BSC20 | Df(3L)BSC20, st[1] ca[1]/TM6B, Tb[1] | 76A7--B1;76B4--5 | |
| | 3617 | Df(3L)kto2 | Df(3L)kto2/TM6B, Tb[+] | 76B1--2;76D5 | |
| | 8088 | Df(3L)ED4858 | w[1118]; Df(3L)ED4858, P{w[+mW.Scer \FRT.hs3]=3'.RS5+3.3'}ED4858/TM2 | 76D3;77C1 | 3L:19888473;20394920 |
| | 27917 | Df(3L)BSC839 | w1118; Df(3L)BSC839/TM6C, Sb1 cu1 | 77B4;77C6 | 3L:20313247;20486308 |
| | 27369 | Df(3L)BSC797 | w[1118]; Df(3L)BSC797/TM6C, Sb[1] cu[1] | 77C3;78A1 | 3L:20445923;20942833 |
| | 25116 | Df(3L)BSC553 | w[1118]; Df(3L)BSC553/TM6C, Sb[1] | 78A2;78C2 | 3L: 20984731--20985064;21219092 |
| | 24923 | Df(3L)BSC419 | w[1118]; Df(3L)BSC419/TM6C, Sb[1] cu[1] | 78C2;78D8 | 3L:21218032;21597878 |
| | 8101 | Df(3L)ED4978 | w[1118]; Df(3L)ED4978, P{w[+mW.Scer \FRT.hs3]=3'.RS5+3.3'}ED4978/TM6C, cu[1] Sb[1] | 78D5;79A2 | 3L:21526907;21873785 |
| | 9700 | Df(3L)BSC223 | w[1118]; Df(3L)BSC223/TM6C, Sb[1] cu[1] | 79A3;79B3 | 3L: 21909520--21909525;22078536 |
| | 24955 | Df(3L)BSC451 | w[1118]; Df(3L)BSC451/TM6C, Sb[1] cu[1] | 79B2;79F5 | 3L: 22069195;22684788--22684831 |
| | 8089 | Df(3L)ED230 | w[1118]; Df(3L)ED230, P{w[+mW.Scer \FRT.hs3]=3'.RS5+3.3'}ED230/TM6C, cu[1] Sb[1] | 79C2;80A4 | 3L:22127751;22827471 |
| | 9226 | Df(3R)ED5100 | w[1118]; Df(3R)ED5100, P{w[+mW.Scer \FRT.hs3]=3'.RS5+3.3'}ED5100/TM6C, cu[1] Sb[1] | 81F6;82E7 | 3R:22995;912807 |
| | 8967 | Df(3R)ED5147 | w[1118]; Df(3R)ED5147, P{w[+mW.Scer \FRT.hs3]=3'.RS5+3.3'}ED5147/TM6C, cu[1] Sb[1] | 82E7;83A1 | 3R:912842;1193526 |
| | 8965 | Df(3R)ED5156 | w[1118]; Df(3R)ED5156, P{w[+mW.Scer \FRT.hs3]=3'.RS5+3.3'}ED5156/TM6C, cu[1] Sb[1] | 82F8;83A4 | 3R:1090655;1284574 |
| | 26533 | Df(3R)BSC681 | w1118; Df(3R)BSC681, P+PBac{XP3.RB5} BSC681/TM6C, Sb1 cu1 | 83E2;83E5 | 3R:2111067;2206257 |
| | 25077 | Df(3R)BSC549 | w[1118]; Df(3R)BSC549/TM6C, Sb[1] | 83A6;83B6 | 3R:1328526;1442413 |
| | 7443 | Df(3R)BSC47 | Df(3R)BSC47, st[1] ca[1]/TM3, P{w[+m*]=Ubx-lacZ.w[+]}TM3, Sb[1] | 83B7--C1;83C6--D1 | |
| | 1990 | Df(3R)Tpl10 | Df(3R)Tpl10, Dp(3;3)Dfd[rv1], kni[ri-1] Dfd[rv1] p[p] Doa[10]/TM3, Sb[1] | 83C1--2;84B1--2 | |
| | 8685 | Df(3R)ED7665 | w[1118]; Df(3R)ED7665, P{w[+mW.Scer \FRT.hs3]=3'.RS5+3.3'}ED7665/TM6C, cu[1] Sb[1] | 84B4;84E11 | 3R:2916249;3919805 |
| | 24970 | Df(3R)BSC466 | w1118; Df(3R)BSC466/TM6C, Sb1 cu1 | 84E1;85A10 | 3R:3657392;4573406 |
| | 9215 | Df(3R)ED5495 | w[1118]; Df(3R)ED5495, P{w[+mW.Scer \FRT.hs3]=3'.RS5+3.3'}ED5495/TM6C, cu[1] Sb[1] | 85F16;86C7 | 3R:5996223;6712482 |
| The 2nd screening | 25724 | Df(3R)BSC633 | w1118; Df(3R)BSC633/TM6C, cu1 Sb1 | 84B2;84C3 | 3R:2906110;2949098 |
| | 7625 | Df(3R)Exel6146 | w[1118]; Df(3R)Exel6146, P{w[+mC]=XP-U} Exel6146/TM6B, Tb[1] | 84C8;84D9 | 3R:2988409;3317319 |
| | 9698 | Df(3R)BSC221 | w[1118]; Df(3R)BSC221/TM6B, Tb[+] | 84C1;84D2 | 3R:2933489;3037519 |
| | 24927 | Df(3R)BSC423 | w1118; Df(3R)BSC423/TM6C, Sb1 cu1 | 84D1;84D5 | 3R:3012954;3222044 |
| | 9076 | Df(3R)ED5223 | w[1118]; Df(3R)ED5223, P{w[+mW.Scer \FRT.hs3]=3'.RS5+3.3'}ED5223/TM6C, cu[1] Sb[1] | 84D9;84E11 | 3R:3317426;3919805 |
| | 25017 | Df(3R)BSC513 | w1118; Df(3R)BSC513/TM6C, Sb1 cu1 | 84D9;84F6 | 3R:3356396;4076143 |
| | 26581 | Df(3R)BSC729 | w1118; Df(3R)BSC729, P+PBac{XP3.RB5} BSC729/TM6C, Sb1 cu1 | 84D14;84F5 | 3R:3575809;4069851 |
